# Identification of *HMX1* target genes: A predictive promoter model approach

**Published:** 2013-08-06

**Authors:** Arnaud Boulling, Linda Wicht, Daniel F. Schorderet

**Affiliations:** 1 Institute for Research in Ophthalmology, Sion, Switzerland; 2 School of Life Sciences, Federal Institute of Technology (EPFL), Lausanne, Switzerland; 3 Department of Ophthalmology, University of Lausanne, Lausanne, Switzerland

## Abstract

**Purpose:**

A homozygous mutation in the H6 family homeobox 1 (*HMX1*) gene is responsible for a new oculoauricular defect leading to eye and auricular developmental abnormalities as well as early retinal degeneration (MIM 612109). However, the *HMX1* pathway remains poorly understood, and in the first approach to better understand the pathway’s function, we sought to identify the target genes.

**Methods:**

We developed a predictive promoter model (PPM) approach using a comparative transcriptomic analysis in the retina at P15 of a mouse model lacking functional *Hmx1* (*dmbo* mouse) and its respective wild-type. This PPM was based on the hypothesis that *HMX1* binding site (HMX1-BS) clusters should be more represented in promoters of *HMX1* target genes. The most differentially expressed genes in the microarray experiment that contained HMX1-BS clusters were used to generate the PPM, which was then statistically validated. Finally, we developed two genome-wide target prediction methods: one that focused on conserving PPM features in human and mouse and one that was based on the co-occurrence of HMX1-BS pairs fitting the PPM, in human or in mouse, independently.

**Results:**

The PPM construction revealed that sarcoglycan, gamma (35kDa dystrophin-associated glycoprotein) (*Sgcg)*, teashirt zinc finger homeobox 2 (*Tshz2)*, and solute carrier family 6 (neurotransmitter transporter, glycine) (*Slc6a9)* genes represented *Hmx1* targets in the mouse retina at P15. Moreover, the genome-wide target prediction revealed that mouse genes belonging to the retinal axon guidance pathway were targeted by *Hmx1*. Expression of these three genes was experimentally validated using a quantitative reverse transcription PCR approach. The inhibitory activity of *Hmx1* on *Sgcg*, as well as protein tyrosine phosphatase, receptor type, O (*Ptpro)* and *Sema3f*, two targets identified by the PPM, were validated with luciferase assay.

**Conclusions:**

Gene expression analysis between wild-type and *dmbo* mice allowed us to develop a PPM that identified the first target genes of *Hmx1*.

## Introduction

The homeobox (HMX) family of transcription factors is characterized by the presence of a 60-amino acid homeobox domain. Currently, this family contains four members: *HMX1*, *HMX2, HMX3* (also known as *Nkx5–3*, *Nkx5–2*, and *Nkx5–1*, respectively), and sensory organ homeobox 1 (*SOHo1*) [1]. Expression of *HMX1*, *HMX2*, and *HMX3* is highest in the sensory organs, I.E., the eye and inner ear, and in the peripheral and central nervous systems [2,3]. During mouse development, *Hmx1* is expressed in the trigeminal ganglion and in the second branchial arches early as E9.5, and in the dorsal root ganglia at E10.5. Later on*,* *Hmx1* is expressed in the lens, in the neural epithelium of the eye, in the sympathic and vagal nerve ganglia, and in the mesenchyme near the developing ear [4]. More recently, the discovery of an *HMX1* loss-of-function mutation responsible for a new oculoauricular syndrome (MIM 612109) in a Swiss consanguineous family prompted us to evaluate the role of this transcription factor [5].

In 2009, the description of two mutant mice called “dmbo” and “misplaced ears” exhibiting microphthalmia, in addition to ear and cranial malformations, was reported. Mapping and sequencing analyses of these mice revealed a nonsense mutation in the first exon of *Hmx1* in *dmbo* and a frameshift mutation in exon 2 of “misplaced ears” mice [6]. The absence of *Hmx1* protein in *dmbo* was further confirmed in a study showing that *Hmx1* was required for the normal development of somatosensory neurons in the geniculate ganglion [7]. Moreover, a *dmbo* rat strain with a similar phenotype and a deletion in an ancient distal putative enhancer of *Hmx1* was described recently [8]. All these rodent mutants underline the prominent role of *Hmx1* in eye development and represent good models.

Despite these recent advances, the role of *Hmx1* in transcriptional regulation remains widely unknown. A major challenge in deciphering the *Hmx1* pathway involved in eye development is identifying target genes. However, this represents a difficult task as no *HMX1* chromatin immunoprecipitation-grade antibody seems to exist in the mouse. Therefore, we constructed a predictive promoter model (PPM). This approach is based on the analysis of differentially co-expressed genes between two different biologic states and represents a powerful tool as was recently shown [9]. In our case, we used a comparative transcriptomic analysis between retinas at postnatal day 15 (P15) of wild-type (WT) and *dmbo* mice. Basically, a promoter model is defined as a framework of two or more transcription factor binding sites (TFBSs) with a defined distance range and strand orientation. In a given promoter, a functional pattern involving multiple TFBSs is called a *cis-*regulatory module (CRM). CRMs represent the next level of organization after individual TFBSs and are often involved in tissue-specific expression (reviewed in [10] and [11]). The promoter model is called predictive when it is based on a functional hypothesis instead of the analysis of experimentally validated TFBSs. In theory, the promoter model represents a specific and flexible structure shared by the promoter of genes belonging to the same pathways.

Despite the lack of knowledge about *Hmx1*, critical information was sufficient to identify specific features about the gene’s target. In fact, Amendt et al. showed that *HMX1* binds to the canonical CAAGTG sequence and acts as a transcriptional antagonist of Nkx2-5, a well-studied transcription factor that recognizes a consensus sequence TNAAGTG overlapping HMX1-BSs [12]. The mouse and rat *ANF* proximal promoters include two validated Nkx2-5-BSs involved in transcriptional activation. Additional sites are located in distal enhancer regions upstream of the transcription start site (TSS) [13-15]. Similar Nkx2–5-BSs clusters have also been observed in the *H15 mid* locus of Drosophila. The functionality of one of them has been demonstrated in cardioblasts [16]. This type of CRM involving multiple similar TFBSs is called homotypic CRMs, or homotypic clusters of TFBSs, and is widely represented in proximal promoters and enhancers of mammals and invertebrates [17]. This observation is particularly true for TFBSs of several TFs, including *Nkx2–5*.

To identify targets of *HMX1*, we developed a PPM based on HMX1-BSs clusters, with analogy to *Nkx2–5*. We report the first *Hmx1* targets in the mouse retina at P15. Moreover, applying our PPM to mouse and human genomes allowed us to identify additional potential target genes involved in embryonic eye development.

## Methods

### Animal handling and tissue isolation

The studies adhered to the Association for Research in Vision and Ophthalmology (ARVO) Statement for the Use of Animals in Ophthalmic and Vision Research and were approved by the Veterinary Service of the State of Valais (Switzerland). WT C57BL/6J mice were obtained from Janvier (Le Genest St Isle, France) and *dmbo* mice from Jackson Laboratory (Bar Harbor, ME). *Dmbo* mice were backcrossed with C57BL/6JWT mice for three additional generations to obtain a homogeneous genetic background and to remove the *Rd1* mutation they unexpectedly carried. All mice were genotyped with polymerase chain reaction analysis of DNA tails. Animals were maintained in a 12 h:12 h light-dark cycle with free access to food and water. Mice were killed by cervical dislocation at P15 or P60, and their eyes were enucleated. Retinas were isolated under a microscope to remove extra retinal tissue and snap-frozen at −80 °C.

### RNA extraction and dosage

Total RNA was individually isolated from each whole retina and purified using the RNeasy minikit (Qiagen, Basel, Switzerland) as described by the manufacturer. RNA quantities were assessed with a NanoDrop ND-1000 spectrophotometer (Thermo Scientific, Wilmington, DE). Four and three different animals for each condition were used for microarray and reverse transcription polymerase chain reaction (RT–PCR), respectively.

### Microarray procedure

RNA quality was assessed using RNA 6000 NanoChips with the Agilent 2100 Bioanalyzer (Agilent, Palo Alto, CA). For each sample, 100 ng of total RNA were amplified using the WT sense strand Target Labeling kit (Affymetrix, Santa Clara, CA); 5.5 µg of the resulting sense cDNA was fragmented with uracil DNA glycosylase (UDG), apurinic/apyrimidic endonuclease 1 (APE 1), and biotin-labeled with terminal deoxynucleotidyltransferase (TdT) using the GeneChip WT Terminal labeling kit (Affymetrix). Affymetrix Mouse Gene 1.0 ST arrays were hybridized with 2.7 µg of biotinylated target for 17 h at 45 °C, washed, and stained according to the protocol described in the Affymetrix GeneChip Expression Analysis Manual (Fluidics protocol FS450_0007). The arrays were scanned using the GeneChip Scanner 3000 7G (Affymetrix), and raw data were extracted from the scanned images and analyzed with the Affymetrix Power Tools software package. All statistical analyses were performed using the free high-level interpreted statistical language R (version 2.12.1) and the Bioconductor package limma (version 3.6.9). Hybridization quality was assessed using the Expression Console software (Affymetrix). Normalized expression signals were calculated from Affymetrix CEL files using the RMA normalization method. Differential hybridized features were identified using Bioconductor package “limma” that implements linear models for microarray data [18]. The p values were adjusted for multiple testing with Benjamini and Hochberg’s method to control for the false discovery rate (FDR) [19]. Probe sets showing at least 1.2-fold change and a FDR<0.1 were considered significant. Gene expression data have been deposited in GEO (GSE47002).

### Functional annotation of microarray data

Differentially expressed genes (FDR<0.1) were annotated in accordance with the Gene Ontology (GO) classification system. GO terms were classified into categories related to molecular function, cell component, and biologic process to assess the statistical enrichment of differentially expressed genes in these categories compared with the full mouse genome. Annotation and statistical calculation were realized using the DAVID algorithm [20,21]. In addition, the MetaCore software from GeneGo was used to highlight the most relevant GeneGO process networks. Each process represents a preset network of protein interactions characteristic of the process. For the DAVID and MetaCore enrichment analyses, only results with a p value <0.1 were considered. MetaCore and DAVID use a hypergeometric model to determine the significance of the enrichment.

### Predictive promoter model construction and validation

All sequences were collected via the UCSC Main Table Browser of the online Galaxy Platform (https://main.g2.bx.psu.edu/). We used the July 2007 (NCBI37/mm9) and February 2009 (GRCh37/hg19) genome assemblies’ versions, and genes absent in the refGene table were retrieved in GenBank and checked manually. All gene accession numbers related to the genes cited in the article are summarized in Appendix 1. [-250;+250] region selection and motif combinations analyses in the (+) and (-)-training sets were obtained from Galaxy. The (-)-training set was constituted by random selection of 2,000 RefSeq gene promoter sequences. Finally, statistical validation of the model was performed with Fisher’s exact test. PPM specificity and sensitivity were calculated with MedCalc. Cell-specific expression levels of *Hmx1*, sarcoglycan, gamma (35kDa dystrophin-associated glycoprotein) (*Sgcg*), teashirt zinc finger homeobox 2 (*Tshz2*), and solute carrier family 6 (neurotransmitter transporter, glycine) (*Slc6a9*) were retrieved in the gene expression profile database [22].

### Predictive promoter model–based genome-wide target predictions

All the [-250,+200] sequences fitting the PPM were selected from human and mouse RefSeq databases. PPM-based selection focused on the conserved HMX1-BS pairs was realized by crossing the human and mouse previously obtained selections. Sequences containing more than two HMX1-BSs were analyzed to achieve the target genes selection based on the co-occurrence of HMX1-BS pairs. Axon guidance pathway enrichment analysis was based on the KEGG database and performed for the mouse predicted target selection obtained with the PPM co-occurrence based method. The statistical enrichment was assessed against the full mouse genome by considering the 25,504 unique genes of the ccds table, with the χ^2^ and Yates' correction test.

### Quantitative rt-PCR

Reverse transcription was performed with 500 ng RNA, 25 ng/µl OligodT, 1 mM each dNTP, 10 mM dithiothreitol, 2 U/µl RNaseBlock, and 1 µl AffinityScript (Agilent) in a final volume of 20 µl at 42 °C for 1 h. The reaction was then maintained at 70 °C for 15 min, and the cDNA obtained was 1:10 diluted. Quantitative PCR was performed in a 25-µl mixture containing 12.5 µl of FastStart Universal SYBR Green Master (ROX; Roche, Basel, Switzerland), 10 µl of diluted cDNA, and 0.3 µM of primer pairs (Appendix 2). The PCR program had an initial denaturation at 95 °C for 10 min, followed by 40 cycles of denaturation at 95 °C for 30 s, annealing at 55 °C for 1 min, and extension at 72 °C for 1 min. All PCRs were realized in triplicate. Transcript levels were normalized using the *Gapdh* housekeeping gene and analyzed with the Student *t* test. All qPCR efficiencies were calculated from the slope of a standard dilution curve to allow relative comparison of gene mRNA levels.

### Immunohistochemistry

Enucleated eyes were fixed for 45 min at 4 °C in 4% paraformaldehyde and were cryoprotected by 30% sucrose. The eyes were then embedded in freezing compound (30% Albumin/3% gelatin in 1x phosphate-buffered saline (PBS): 154 mM NaCl, 1 mM KH_2_PO_4_, 3 mM Na_2_HPO_4_ heptahydrate) and vertically sliced 10 μm thick in a cryostat. Sections were washed three times with PBS, treated with blocking buffer (2% native goat serum containing 0.2% Triton X-100) at room temperature for 10 min, and left overnight at 4 °C with the anti-SGCG primary antibody (Proteintech, Chicago, IL) diluted 1:100. Controls were prepared by omitting the primary antibody during the incubation. The following morning, sections were rinsed three times with PBS, blocked for 10 min, and incubated at room temperature for 1 h with Alexa Fluor 594 goat antirabbit immunoglobulin (Invitrogen, Zug, Switzerland) secondary antibody diluted 1:1,500. After three additional washings, the sections were stained with 4',6-diamidino-2-phenylindole dihydrochloride diluted 1:1,500 for 10 min at room temperature, washed three times again, and mounted with Citifluor AF1 (Citifluor Ltd, Leicester, UK). The stained slides were imaged on a Zeiss microscope, and image analysis was performed using the ZEN lite 2011 software (Zeiss, Zürich, Switzerland).

### Construction of reporter and expression plasmids

PCR primer pairs used for molecular cloning were designed to generate an amplicon spanning the TSS of *Sgcg*, *Sema3f*, and *Ptpro* and to carry all the HMX1-BS identified in the proximal promoter region. All promoter regions were amplified with the *PfuUltra* High-Fidelity DNA Polymerase (Agilent) according to the manufacturer’s instructions. Mouse *Sgcg*, *Sema3f*, and *Ptpro* promoter regions were amplified using the following primer pairs carrying MluI and XhoI restriction sites (underlined), with indicated annealing temperatures (Ta): 5′-GCG CAC GCG TCA AAG ACA CGT CAG CCT CAG-3′ and 5′-GCG CCT CGA GGA AAC GCT GTA CCT ATC TGA TTT ACA-3′ (*Sgcg*, Ta=61 °C), 5′-GCG CAC GCG TGC AAG AGT GTA TGG GGA AGG-3′ and 5′-GCG CCT CGA GCA GGC CTC TCA GCA GGTG-3′ (*Sema3f*, Ta=63 °C), 5′-GCG CAC GCG TCA TGG AAA TCG TTG CTT GTG-3′ and 5′-GCG CCT CGA GCG GCG TTG TTT AAT GGC TAA-3′ (*Ptpro*, Ta=60 °C). Amplified DNA fragments were inserted into the pGL3-basic vector with the XhoI and MluI restriction enzymes to produce pGL3-*Sgcg*, pGL3-*Sema3f*, and pGL3-*Ptpro* reporter constructs. Before the cloning step, the CAAGTG site located just upstream of the multiple cloning site in the pGL3-basic vector was converted to TAATCA by site-directed mutagenesis. The *Hmx1* mouse cDNA was amplified with *PfuUltra* High-Fidelity DNA Polymerase using 5′-ATG CCG GAT GAG CTG ACC G-3′ and 5′-TCA CAC TAG CCC CGG CAT C-3′ primers (Ta=60 °C), and then inserted into the pcDNA3.1 vector (pcDNA3.1-Hmx1) with the pcDNA3.1/V5-His TOPO TA Expression Kit (Invitrogen).

### Cell culture and transfection

Mouse neuroblastoma cells (aka Neuro-2a or N2a) were grown in Dulbecco’s Modified Eagle Medium (DMEM; PAA, Cölbe, Germany) supplemented with 10% fetal bovine serum and 100 µg/ml Normocin (Invivogen, Toulouse, France). Twenty-four hours before transfection, 200,000 cells/well were seeded in 12-well plates. For one transfection, 900 ng of one pGL3 reporter construct, 900 ng of pcDNA3.1-Hmx1 or empty pcDNA3.1, and 300 ng of pCMV-Beta-Gal control plasmid were mixed together with 4 µl jetPEI (Polyplus, Illkirch, France) and dropped onto the cells.

### Luciferase reporter gene assay

Forty-eight hours after transfection, cells were rinsed with PBS and lysed with 100 µl potassium phosphate buffer (100 mM K_2_HPO_4_, pH7.8, 0.2% Triton X-100). After centrifugation, 5 µl supernatant from each sample were added to 20 µl Firefly luciferase reagent. In parallel, 10 µl supernatant from each sample were added to 100 µl β-galactosidase reagent. The relative luciferase activity was determined by dividing the luminescence of Firefly luciferase activity by that of the cotransfected β-galactosidase activity. The experiment was performed three times for each reporter construct, and transfections were realized in triplicate for each experiment. The significance between the luciferase activity of each reporter construct cotransfected with pcDNA3.1-Hmx1 and with pcDNA3.1 was then assessed for significance with the Student *t* test.

### Statistical analysis

The statistical tests used in this study are detailed at the end of the microarray procedure, functional annotation of microarray data, PPM construction and validation, quantitative reverse transcription PCR, and luciferase reporter gene assay sections.

## Results

### Comparative transcriptomic analysis

**Figure 1:**
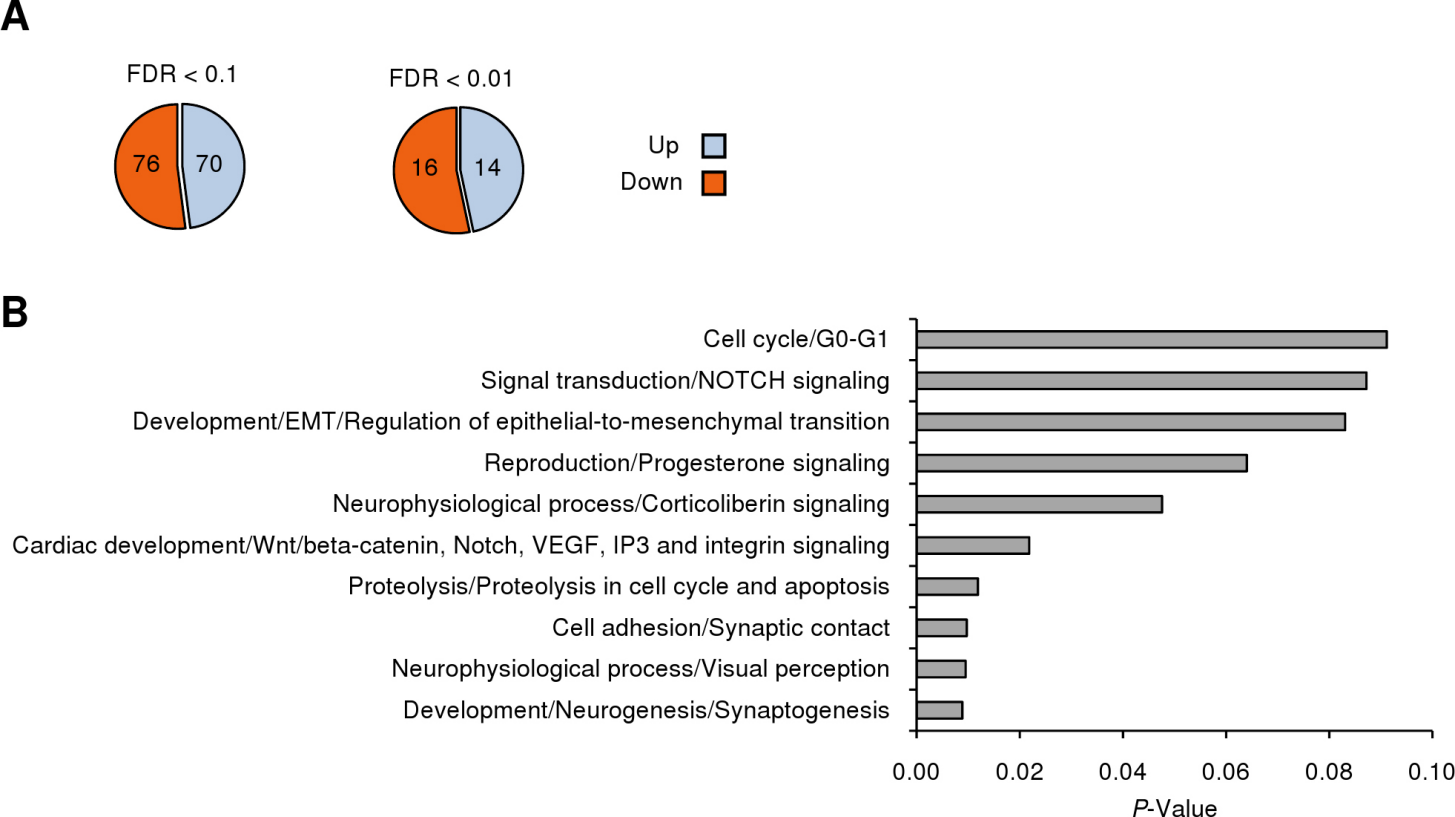
Summary of microarray results. **A**: Up- and downregulated genes with a fold-change >1.2 and a FDR<0.1 or <0.01. **B**: MetaCore GeneGO Process Networks enrichment analysis of the FDR<0.1 group of differentially expressed genes.

The comparative transcriptomic analysis of the mouse retina between the *dmbo* and WT C57BL/6J mice was realized at P15 to avoid killing pregnant *dmbo* mice and to obtain a sufficient amount of tissue. The retina is still developing at this age and is always expressing *Hmx1*. The analysis showed 146 differentially expressed genes (70 up and 76 down) with a FDR<0.1 and at least 1.2-fold change (Figure 1A, Appendix 3, Appendix 4). Thirty of these genes were highly confident and had a FDR<0.01 (14 up and 16 down). Analysis of the 146 differentially expressed genes with the MetaCore software from GeneGo showed ten enriched GeneGO process networks with p<0.1 (Figure 1B). The first three ranked processes are the synaptogenesis (p=0.008879), the visual perception (p=0.009481), and the synaptic contact (p=0.009713). In another approach, the DAVID software allowed us to classify these genes into GO categories, showing that nine of them were significantly enriched with p<0.1 in molecular function, three in cell component, and 20 in biologic process categories (Appendix 5). All of the enriched molecular function categories were related to ion and vitamin binding or transmembrane transport, and all the enriched cell component categories were related to cell projection terms as axoneme and cilium. Enriched biologic process categories were more numerous and diversified and concerned, for example, organelle localization or metal ion homeostasis.

### Hmx1 target promoter model construction and validation

**Figure 2:**
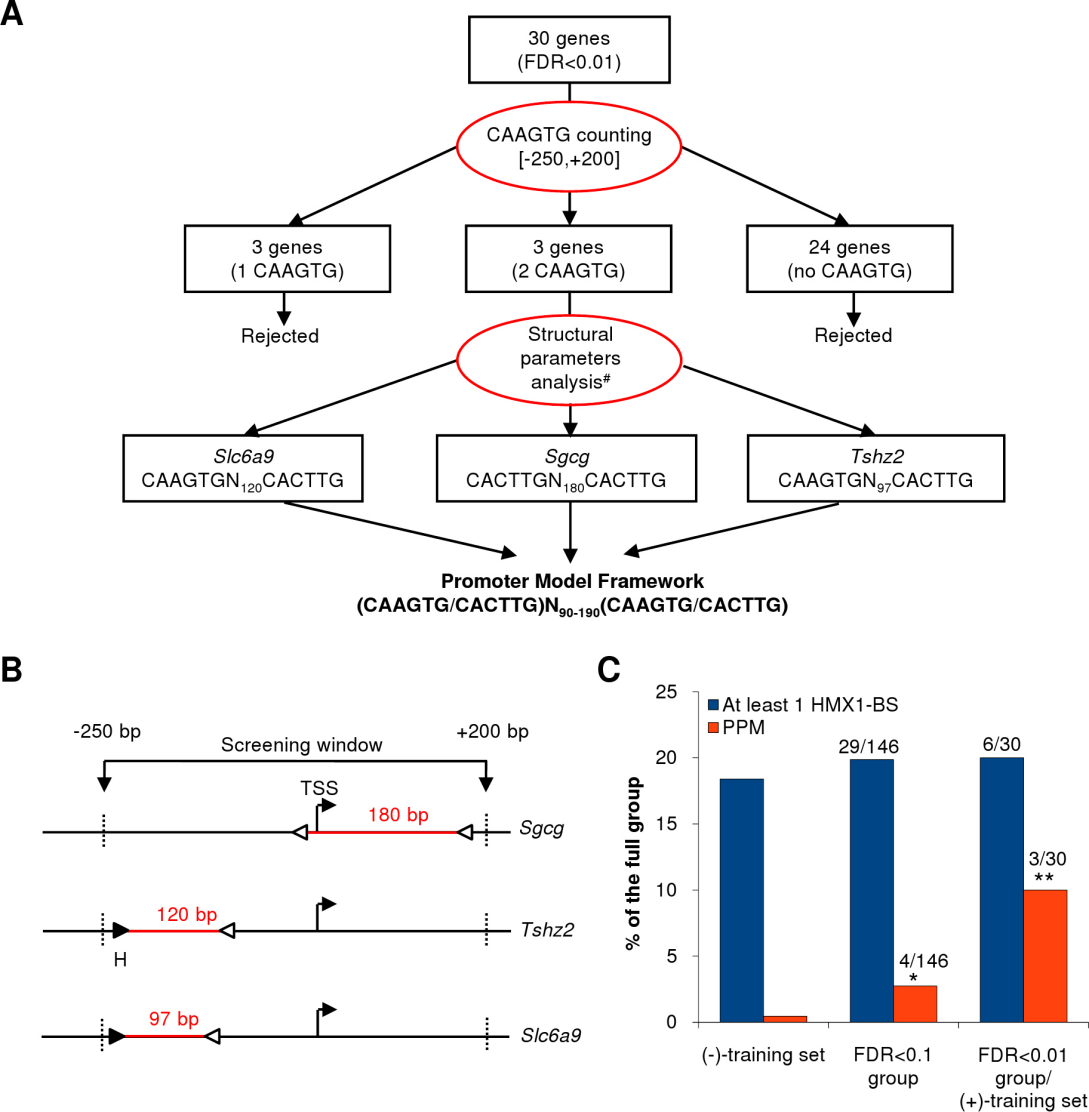
Elaboration and statistical validation of an Hmx1 predictive promoter model. **A**: Flowchart of the predictive promoter model (PPM) construction. #Location of CAAGTG motifs within proximal promoter or 5'UTR region is given in (**B**). **B**: Details of *Sgcg*, *Tshz2*, and *Slc6a9* promoter structure. Forward (CAAGTG) and reverse (CACTTG) *HMX1* binding sites (HMX1-BSs) are symbolized by black and white triangles, respectively. The H letter indicates an HMX1-BS that is strictly conserved in human. **C**: Statistical validation of the PPM. Columns represent the percentage of promoters carrying at least 1 HMX1-BS or fitting the PPM in each group. Promoter count details are indicated for the false discovery rate (FDR)<0.1 group and the (+)-training set (FDR<0.01 group). *p<0.01, **p<0.001.

As explained above, we hypothesized that multiple *Hmx1* sites could form CRMs and act in synergy, as observed for *Nkx2–5* [17]. In this regard, we postulated that the number of motifs could play a role in the transcriptional regulation of *Hmx1* targets. To elaborate our predictive promoter model, we used the promoter sequences of the most confident differentially expressed genes (FDR<0.01 group) and considered the number of CAAGTG motifs present. We used a screening window ranging in size from −250 to +200 nucleotides (nt) around the TSS. This window was based on the size of the proximal promoter and the approximate median size of the eukaryote 5′ untranslated region (UTR), two regions known to be enriched in TFBS [23,24]. The CAAGTG motifs located in the [-250,+200] interval were counted. Three of the 30 genes contained two motifs, three contained one motif, and the last 24 did not contain any motif. The three genes containing two motifs, considered theoretical *Hmx1* targets, were used to generate a framework (Figure 2A–B) that should correspond to a feature specific for *Hmx1* target promoters. The orientation of the motifs was disregarded, but we considered the space between the two motifs and kept a distance range spanning from 90 to 190 nt. Unexpectedly, only one HMX1-BS in the *Tshz2* promoter was strictly conserved between human and mouse.

This theoretical promoter model is predictive and must be statistically validated, as it is based on arbitrary criteria. Therefore, we calculated the enrichment of this particular feature between the FDR<0.01 group (used as the positive (+)-training set) and a control group of random promoters (used as the negative (-)-training set). We observed a 22.2-fold significant enrichment (p=0.0006) of the PPM specific feature in the (+)-training set compared to the (-)-training set within the [-250,+200] region (Figure 2C). This enrichment was also observed in the FDR<0.1 group but to a lesser extent (p=0.0091). There was no significant enrichment when the genes containing at least one motif were considered. From these data, we concluded that the high enrichment of this particular feature in the (+)-training set validated our promoter model and supported the idea that *Sgcg*, *Tshz2*, and *Slc6a9* were direct *Hmx1* targets.

To complete our approach, we also tried to build a PPM with lower specificity and higher sensitivity. For this, we replaced the canonical sequence CAAGTG with the minimal core motif CAAG also able to bind *HMX1* but with a lower affinity [12]. We generated a low specific PPM (LS-PPM) and a very low specific PPM (VLS-PPM) fitting the same distance and orientation criteria than the initial PPM, but carrying one or two CAAG in place of the canonical HMX1-BS, respectively (Table 1). Both retrieved a better rate of positive genes but showed low specificity (1.37- and 1.01-fold enrichment for LS-PPM and VLS-PPM, respectively). These two PPMs with lower specificity were not reused for the following analyses.

**Table 1:** Sensitivity and specificity of PPM-based selection methods.

Characteristics of PPMs	PPM (2 HMX1-BS)	LS-PPM (1 HMX1-BS + 1 CAAG)	VLS-PPM (2 CAAG)
Predicted targets in the (-)-training set (%)	0.5	14.7	59.3
Predicted targets in the (+)-training set (%)	10.0	20.0	63.3
Fold-enrichment; *P* value (if <0.05)	22.2; 0.0006	1.4	1.0
Sensitivity	10.0% (95% CI:2.2% - 26.6%)	20.0% (95% CI:7.8% - 38.6%)	63.3% (95% CI:43.9% - 80.1%)
Specificity	99.6% (95%CI:99.2% - 99.8%)	85.4% (95% CI:83.7% - 86.9%)	40.8% (95% CI:38.6% - 42.9%)

LS-PPM, Low specific PPM; VLS-PPM, Very low specific PPM. 95% CI, 95% confidence interval. CAAG, minimal core motif able to bind *HMX1*.

### Characterization of *Sgcg*, *Tshz2*, and, *Slc6a9* expression

**Figure 3:**
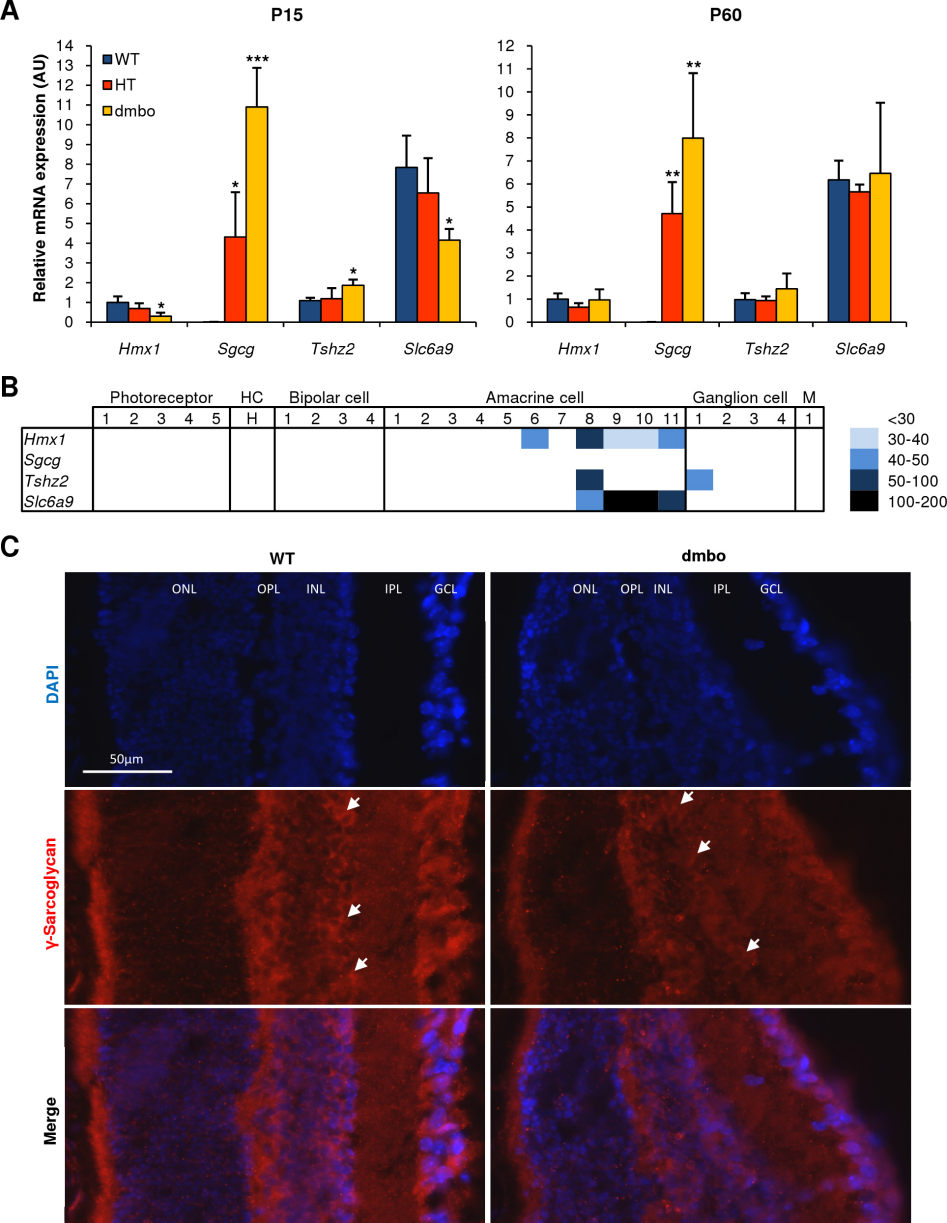
Characterization of *Sgcg*, *Tshz2*, and *Slc6a9* expression. **A**: Quantitative reverse transcription polymerase chain reaction (qPCR) analysis of *Sgcg*, *Tshz2*, and *Slc6a9* expression in wild type (WT), heterozygous (HT), and *dmbo* whole retina at P15 and P60. The significance of the differences between the WT and HT or *dmbo* mean mRNA expression levels were determined by three independent experiments done in triplicate. All qPCR efficiencies were above 1.96 with a Pearson’s r above 0.99. The mRNA levels are expressed as a ratio of the H6 homeobox family 1 (*Hmx1*) WT level, in P15 and P60 experiments, independently. Bars, SD; *p<0.01, **p<0.05, ***p<0.001. AU, arbitrary units. **B**: Heatmap representation of *Hmx1*, *Sgcg*, *Tshz2*, and *Slc6a9* expression in the different cell types of the mouse retina according to the gene expression profile database [22]. **C**: Immunostaining of γ-Sarcoglycan in the WT and *dmbo* retina at P15. White arrows indicate an accumulation of γ-Sarcoglycan at the border delimitating the INL and the IPL. DAPI, 4',6-diamidino-2-phenylindole dihydrochloride staining; Merge, overlap between DAPI and γ-SG immunostaining. ONL, Outer nuclear layer; OPL, Outer plexiformlayer; INL, Inner nuclear layer; IPL, Inner plexiform layer; GCL, Ganglion cell layer.

To minimize the possibility that *Sgcg*, *Tshz2*, and *Slc6a9* were false positive targets of *Hmx1*, we confirmed their level of deregulation between eyes from *dmbo* and WT mice, and checked for colocalization with *Hmx1*. The retina is made of many different cell types with specific expression profiles. We therefore assessed the expression of *Hmx1, Sgcg*, *Tshz2*, and *Slc6a9* with qPCR (Figure 3A) and looked for their precise cell subtype expression in the mouse retina, according to an online gene expression profile database [22] (Figure 3B). Transcript quantification confirmed that *Sgcg* and *Tshz2* were overexpressed in *dmbo* at P15, whereas *Slc6a9* was underexpressed. This deregulation tended to disappear at P60 for *Tshz2* and *Slc6a9*, but remained extremely high for *Sgcg*. As expected, the inspection of a retina-specific database revealed a strong overlap of *Hmx1, Slc6a9*, and *Tshz2* expression in the glycinergic amacrine cells. *Sgcg* expression was not detected in the microarray database probably due to the weak level of mRNA, as shown with qPCR. However, the γ-sarcoglycan protein encoded by *Sgcg* was detected with immunohistochemistry in the ganglion cell (GCL), the inner plexiform (IPL), the inner nuclear (INL), and the outer plexiform layers (OPL; Figure 3C), as already shown by Fort et al. [25]. No difference in protein expression of γ-sarcoglycan was observed between the WT and *dmbo* samples (data not shown). The immunohistochemistry staining was higher in some cells of the INL exhibiting a disposition pattern characteristic of the amacrine cells, at the delimiting border between the INL and the IPL. This result was in accordance with colocalization of *Sgcg* and *Hmx1* expression.

### Predictive promoter model–based genome-wide screening for HMX1 putative targets

Our model was based on a comparative transcriptomic analysis realized in the mouse retina at P15. However, *Hmx1* is highly expressed in the mouse eye as early as E10.5 suggesting an important role in development. Assuming that the PPM we developed was specific for *HMX1* targets (with 0.45% versus 10% representation in the (-) and (+)-training sets, respectively), we decided to screen the full human and mouse genomes. This global approach should provide an exhaustive view of all putative *HMX1* targets, including those expressed during embryonic eye development. As the first step, we used the PPM to screen mouse and human RefSeq databases via the Galaxy platform. These two databases contained a total of 30,490 and 43,695 sequences respectively, which corresponded to all transcripts of reference, including all isoforms and alternative promoters. We considered for each gene all potential alternative promoters whereas all redundant promoter sequences due to alternative splicing were discarded. The gene accession numbers of all isoforms corresponding to our predicted genes are listed in Appendix 1.

**Figure 4:**
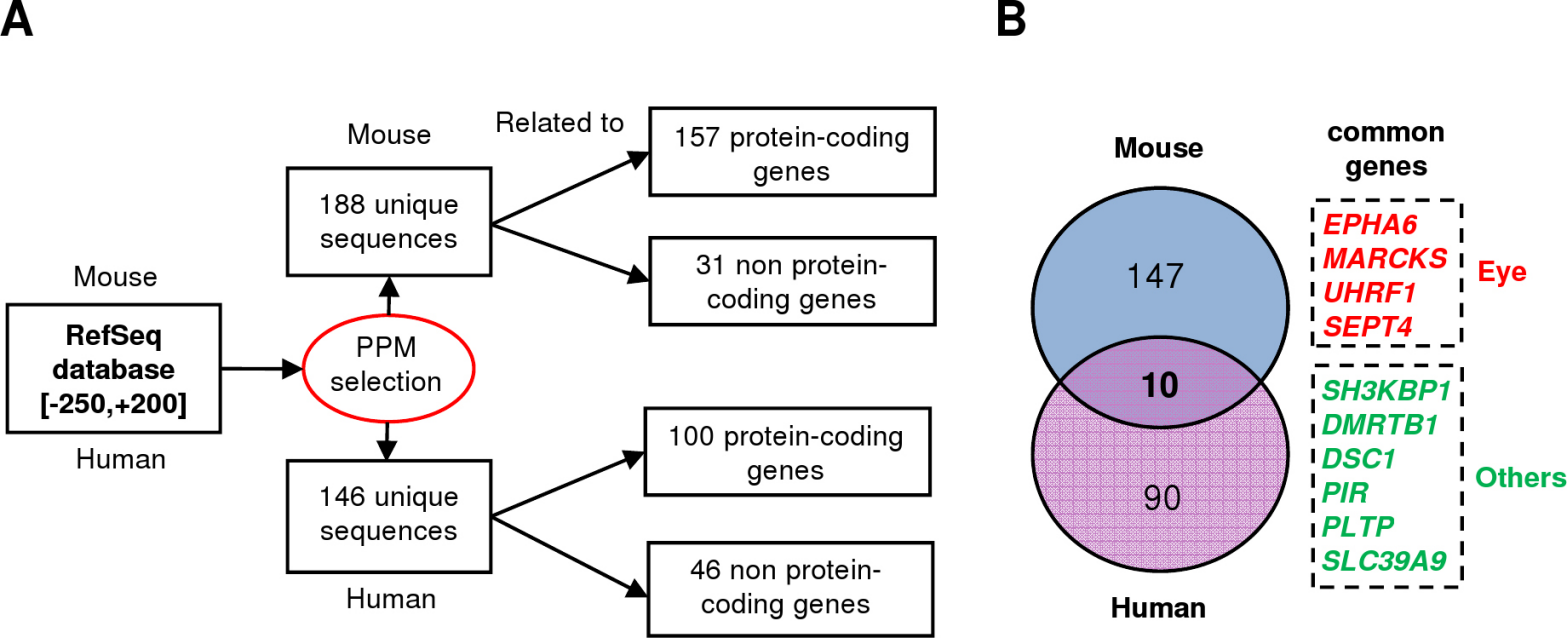
Predictive promoter model–based genome-wide target selection focused on the conserved HMX1-BS pairs. **A**: Flowchart of the predictive promoter model (PPM)-based genome-wide target selection. **B**: Venn diagram illustrating the overlap between human and mouse target selections. Expression localization in the eye is indicated for genes carrying an HMX1-BS pair in both species.

Screening of the full mouse RefSeq database using the PPM within the [-250,+200] region retrieved 157 sequences corresponding to unique protein-coding genes (Figure 4A, Appendix 1). Similarly, the screening of the human genome retrieved 100 sequences corresponding to unique protein-coding genes. This approach allowed us to generate an exhaustive list of all possible *HMX1* targets, but the high number of positive hits resulted in some difficulties with their interpretation. In fact, some of these genes were probably true *HMX1* targets, but several could also be false-positives, representing targets related to TFs with the same binding sites (for example, *HMX3* or *NKX2–5*). To improve the selectivity of the analysis and to focus on the most interesting candidate genes, we decided to add additional filters.

Initially, we developed a PPM approach based on conserving the HMX1-BS pairs between human and mouse. In fact, comparative genomics is one of the usual methods that aimed at discriminating functional TFBSs from irrelevant ones (reviewed in [11]). To maintain relative flexibility, our method was based only on the presence of an HMX1-BS pair and did not implicate a strict conservation of the positions or orientations of the HMX1-BSs between the two species. As already demonstrated, traditional approaches, similar to phylogenetic footprinting, give good predictions but are also likely to miss important conserved regulatory elements [26]. Crossing the two data sets showed that only ten genes contained the PPM features in both species (Figure 4B). These genes were classified according to the localization of their expression.

**Figure 5:**
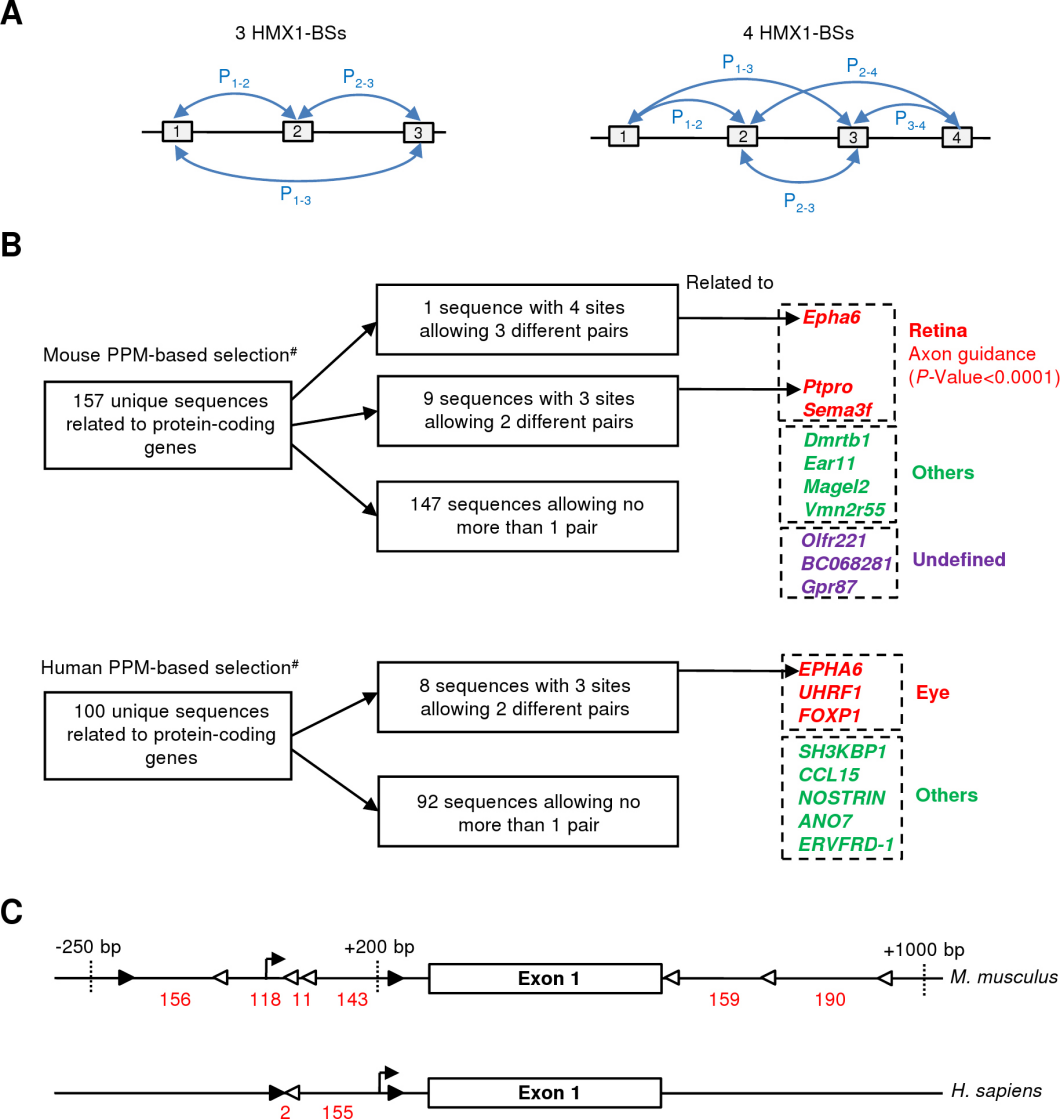
Predictive promoter model–based genome-wide target selection focused on the co-occurrence of HMX1-BS pairs. **A**: Simultaneous possible combinations of HMX1-BS pairs allowed by the predictive promoter model (PPM) with three or four HMX1-BSs. Pairs are identified according to HMX1-BSs numbers. **B**: Flowchart of the mouse and human PPM-based selections filtering according to HMX1-BS pairs counting. ^#^See Figure 4 for details regarding the PPM-based selection process. Expression localization in the retina is indicated for genes carrying multiple HMX1-BS pairs. Significant enrichment in the axon guidance pathway is also indicated (see the text). **C**: Details of human and mouse *EPHA6* structure in the region surrounding the transcription start site (TSS). Forward (CAAGTG) and reverse (CACTTG) HMX1-BSs are symbolized by black and white triangles, respectively. Distances (bp) between HMX1-BSs are indicated in red.

In a second phase, we used another PPM approach based on the cooccurrence of HMX1-BS pairs, driven by the basic idea that increasing the number of HMX1-BS in the promoter region should increase their interaction with *HMX1*. This phenomenon should result in more efficient transcription regulation. To assess this hypothesis, we looked at the HMX1-BS occurrence in all the mouse and human promoter sequences fitting the PPM. We observed that the promoter regions contained a maximum of four HMX1-BSs within the [-250,+200] window. Always in accordance with the PPM criteria, we determined that three sites might form three different homotypic HMX1-BS pairs (P_1–2_,P_1–3_,P_2–3_), even if it is unlikely that all three pairs could be considered at the same time due to the minimum distance range constraint of 90 bp (Figure 5A). Similarly, four sites might lead to a maximum of five combinations (P_1–2_, P_1–3_,P_2–3_, P_2–4_, P_3–4_). A single given site can be involved in multiple combinations. The screening of the mouse genome with this method retrieved nine genes with three sites allowing two different pair combinations, and one gene (Ephrin type-A receptor 6; *Epha6*) with four sites allowing three different pair combinations (Figure 5B). Interestingly, three of these ten genes (*Epha6*, *Ptpro*, and *Sema3f*) are expressed in the retina and are involved in axonal growth repulsion (see discussion). It represents a 56.6-fold enrichment (p<0.0001) in the mouse axon guidance KEGG pathway (mmu04360). *Ptpro* was incorporated within the pathway although this gene was not initially reported in the KEGG database, in spite of *Ptpro*’s role as a guidance cue in retinal neurons [27,28]. Moreover, a deeper examination of *Epha6* showed an additional HMX1-BS at position [+245,+250] and an additional HMX1-BSs cluster fitting the PPM within the first intron (Figure 5C). Among the mouse HMX1-BSs, two were conserved in the human *EPHA6* proximal promoter. One was unique to the human gene. With a similar approach, we identified eight genes with three sites allowing two interactions in the human genome (Figure 5B).

### Experimental validation of several predicted targets with luciferase assay

**Figure 6:**
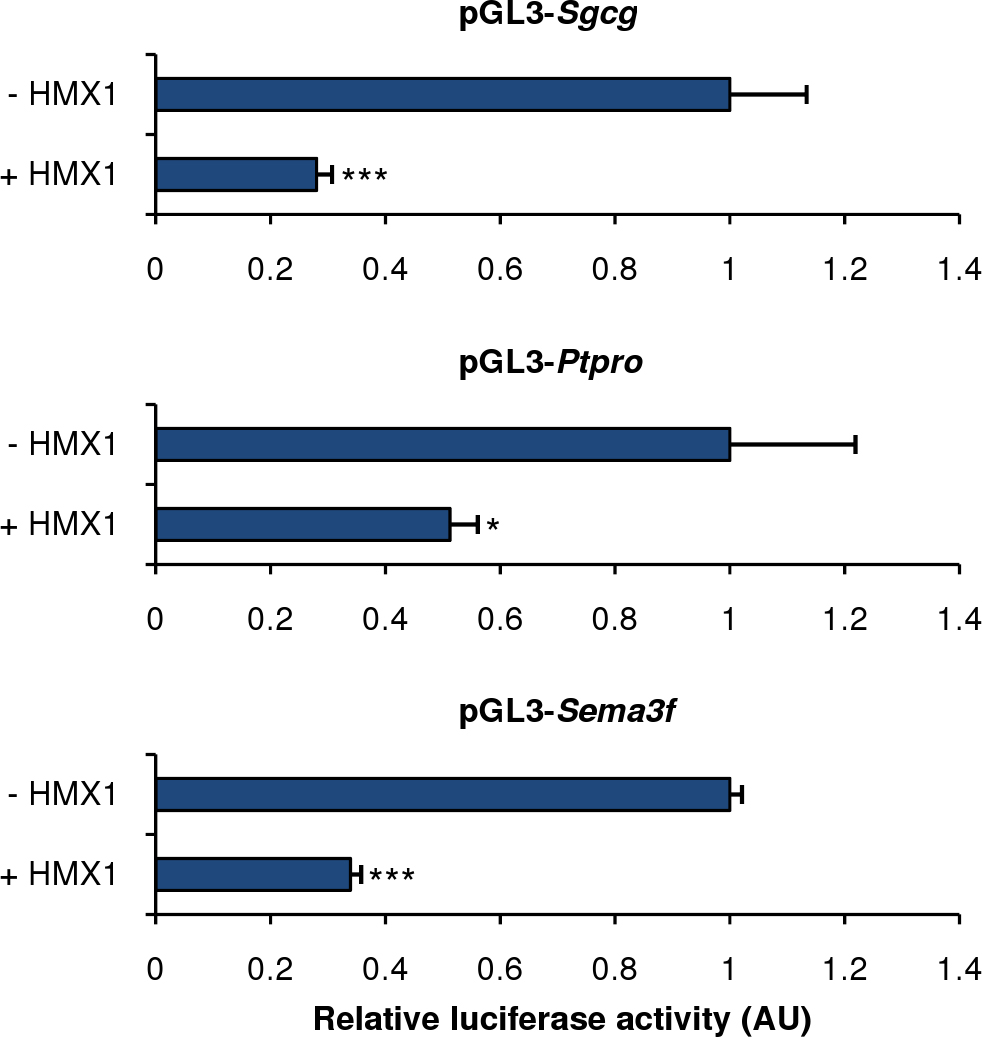
Transcriptional repression of predicted target genes by the H6 homeobox family 1 gene. The pGL3-*Sgcg* (positive control), pGL3-*Sema3f*, and pGL3-*Ptpro* reporter plasmids were cotransfected with the empty pcDNA3.1 or the pcDNA3.1-Hmx1 expression vectors into the N2A cells. For each gene, the activities are shown relative to the pGL3 constructs cotransfected with the control empty pcDNA3.1. Firefly luciferase activities were normalized against the cotransfected β-galactosidase activity. Bars, SD; *p<0.01, ***p<0.001. AU, arbitrary units.

We experimentally validated some of these results with luciferase assays performed in N2A cells. We first assessed the reliability of our system by cotransfecting the pGL3-*Sgcg* positive control reporter construct with the pcDNA3.1-*Hmx1* expression construct. *Hmx1* cotransfection decreased pGL3-*Sgcg* luciferase expression by 71%, which was expected given the *dmbo* qPCR *Sgcg* results (Figure 6). Cotransfection of *Hmx1* repressed pGL3-*Ptpro* and pGL3-*Sema3f* luciferase expression by 50% and 66%, respectively.

## Discussion

The major goal of this study was to identify target genes of *Hmx1* in the mouse retina. The integration of in vitro data from Ament et al. and our in vivo microarray data allowed us to obtain a clear picture of the typical basic structure of an *HMX1* target promoter [12]. In addition, experimental controls concerning transcript amounts, expression colocalization, and luciferase assay strongly supported these findings.

The microarray data yielded the first set of information about the molecular phenotype of the *dmbo* retina. The MetaCore analysis underlined that a lack of HMX1 protein altered synaptogenesis and visual perception, two biologic processes occurring at P15 [29]. At this time, we cannot say whether these observations were directly linked to *Hmx1* loss of activity or if they derived from anterior impairments occurring during eye development.

Then, we used the microarray data to construct a high specific PPM based on HMX1-BS clusters. It revealed that *Sgcg*, *Tshz2*, and *Slc6a9* were *Hmx1* targets in the mouse retina at P15. Using degenerated binding motifs for PPM construction, such as the minimal CAAG core motif, led to higher sensitivity but also to low specificity with no significant enrichment. Such a low specific PPM cannot be used for prediction because the model would probably yield an extremely high number of false positives. However, we thought that *Hmx1* probably binds degenerate motifs in vivo, but no position weight matrix is currently available to perform a better PPM for *Hmx1*. Moreover, the sensitivity of the original PPM is likely underestimated because some of the differential expressions observed for genes belonging to the (+)-training set probably result from secondary events and are not directly linked to *Hmx1*.

The expression of *Hmx1*, *Tshz2*, *Slc6a9*, and probably *Sgcg* was observed in the glycinergic amacrine cells, a cell type that establishes synaptic contacts with rod-driven bipolar cells and play an important role in neurotransmission. *Tshz2* is involved in an axonal growth network in the mouse retina and is expressed in the zebrafish neural retina at 48 h post fertilization [30,31]. *Slc6a9* is specifically expressed in the glycinergic amacrine cells where it plays an important role in glycine uptake, and controls N-methyl-D-aspartic acid receptor coagonist occupancy in the mouse retina [32]. The role of both genes in the retina needs to be further investigated. However, their expression seemed to be totally or partially compensated at P60, suggesting that *Hmx1* does not solely regulate them. The positive deregulation of *Sgcg* in qPCR was impressive (about 1,000-fold), but it did not correlate with a higher amount of proteins in *dmbo* retina. It is likely that *Sgcg* was strongly regulated at the level of translation, which would explain why this dramatic increase in transcripts had no effect on the protein level, as shown for other genes related to cell adhesion [33]. In addition, the overexpressed γ-sarcoglycan could form aggregates that could be degraded in the endoplasmic reticulum, as supported by the proteolysis process enrichment in MetaCore analysis (Figure 1B). The role of the *sgcg* gene in the *dmbo* retinal phenotype remains unclear. Another finding resulting from the microarray analysis was that *Hmx1* could act in vivo as a transcriptional repressor or activator. The first in vitro study conducted by Ament et al. showed only a repressor effect, but their work was done in HeLa cells indicating that cellular context may play a role in mediating *HMX1* activity [12].

**Figure 7:**
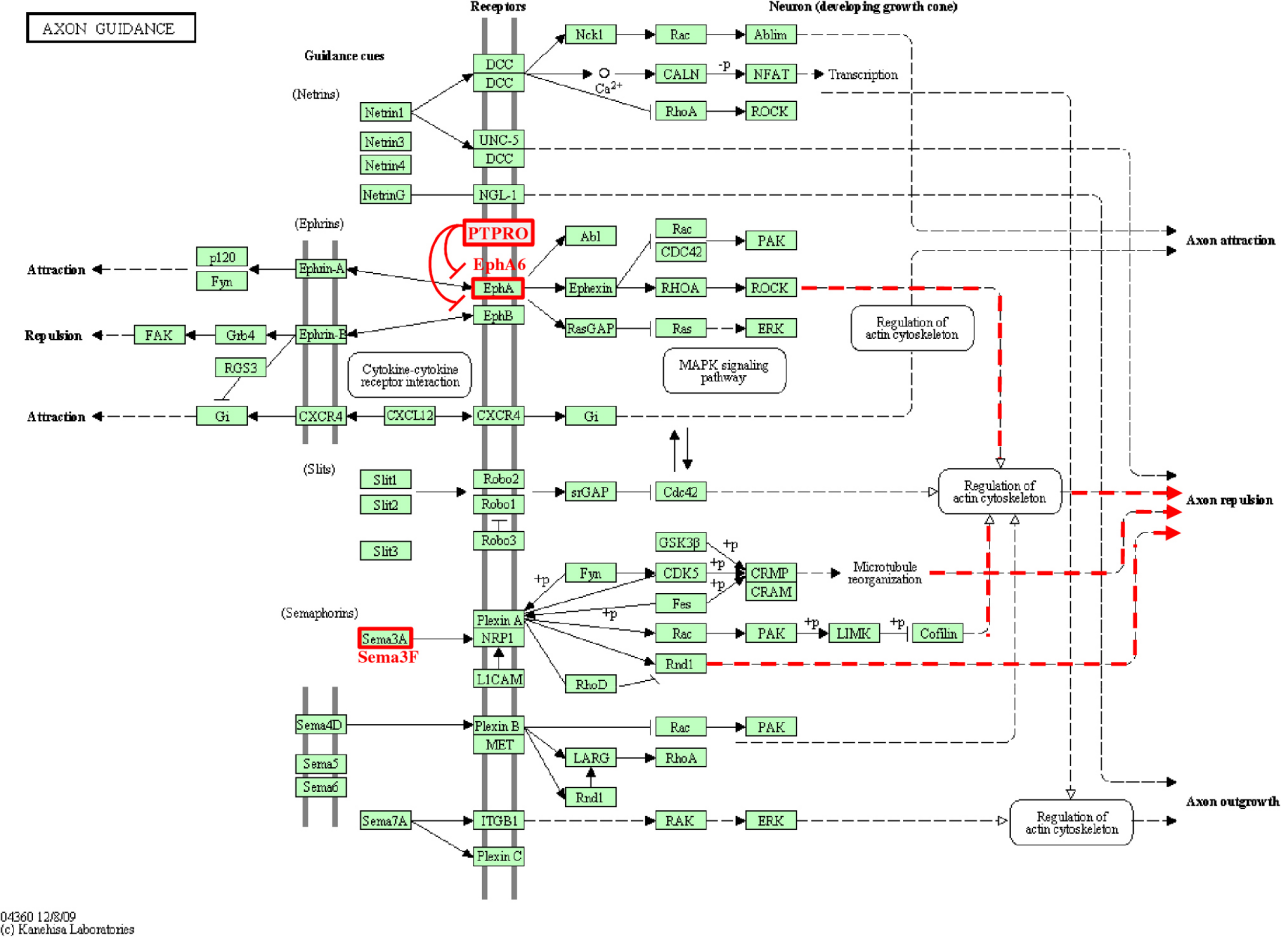
Mouse axon guidance pathway map. Adapted from the mmu04360 KEGG pathway. EphA6, Sema3F, and PTPRO are localized in red in the pathway. Note that PTPRO was originally absent from the map. Outputs of the ephrin and semaphorin pathways are underlined with red dotted lines. Green boxes: genes or gene products; rounded white boxes: connected pathways; solid arrows: direct interactions; dotted arrows: indirect interactions. Normal arrows symbolize activation, headless arrows symbolize inhibition.

The second part of our study consisted of using our PPM to screen the genome and discover other putative targets of *HMX1*. More precisely, we focused our attention on identifying *HMX1* targets that could be involved in eye development. This would help in understanding the bases of the human oculoauricular syndrome caused by *Hmx1* mutation [5]. The first method consisted of using the PPM to screen the RefSeq database of mouse and human. This method retrieved a large but expected number of genes despite the short screening window used. Many of these genes represented interesting candidates (see Appendix 1 for a complete list). To be more selective, we crossed human and mouse selections to keep only genes fitting the PPM in both species. Among the ten genes that we retrieved, four have been reported to be expressed in the eye, during development (*EPHA6*, *MARCKS*, and *UHRF1*), or during adult life (*SEPT4*) [34-37]. In addition, *SH3KBP1* was predicted to be a target of *HMX1* and is highly expressed in Schwann cells, where the gene is regulated by *SOX10* [38]. Interestingly, a recent study showed that a balance between *SOX10* and *HMX1* regulates neuronal versus Schwann cell precursor and melanocyte fates [39]. Our second method based on the co-occurrence of HMX1-BS pairs retrieved ten genes in the mouse genome and eight in the human genome. *EPHA6*, *UHRF1*, and *SH3KBP1* were identified by both methods, increasing the confidence that these three targets were true targets. In the mouse, the *Epha6* promoter showed the highest number of HMX1-BS pair combinations with four sites located within [-250,+200]. A wider examination of the region surrounding its TSS showed additional HMX1-BS clusters fitting the PPM in the proximal promoter and in the first intron. With *Ptpro* and *Sema3f*, *Epha6* belongs to the retinal axon guidance pathway and plays an important role in retinotopic mapping [27,28,34,40,41]. In addition, these three predicted targets occupy key places as inputs of the axon repulsion signaling pathway, supporting a specific and effective action of *Hmx1* in this process (see Figure 7 for more details). Finally, the strong and highly significant enrichment of this pathway in the mouse selection obtained with HMX1-BS pairs co-occurrence based method underlined the likely role of *Hmx1* in establishing retinal topography. The luciferase assay results provided experimental evidences to validate this hypothesis. For a positive control, we first showed that *Hmx1* could act as a repressor and decrease the activity of the *Sgcg* promoter in the N2A cells. This result was expected based on the dramatic increase of *Sgcg* expression in *dmbo* mice. Then, we showed that *Hmx1* represses the activity of *Ptpro* and *Sema3f* promoters. In the future, we will specifically focus our efforts on the functional study of *Epha6,* all the more so as the Ephrin pathway was already considered by Schorderet et al. to be a putative target of *Hmx1* [5]. We also noted that the human selection contained a new interesting gene expressed in the eye, *FOXP1* [42], in addition to *UHRF1* and *EPHA6*. Finally, some of the remaining predicted targets unrelated to the eye could be potential *HMX1*, *NKX2–5*, or *HMX3* targets in other tissues and will need further investigation.

Several recurrent questions about TFBS identification arose from our study. The first concerns the conservation of CRMs between human and mouse. In fact, we observed poor conservation of our PPM between both species, whereas numerous studies showed that evolutionary conserved regions overlap functional regulatory elements (reviewed in [11]). Actually, approaches integrating comparative genomics data succeed to identify CRMs with a high positive predictive value but overlook CRMs specific for a given species. Single-genome bioinformatic approaches are more convenient to solve this problem. In this manner, a study based on the empirical potential energy of TFs revealed that CRMs occur in conserved and non-conserved regions, and about 55% have a poor conservation score [43]. In particular, this study underlined that the less well-conserved CRMs concern genes related to neural activity. It could be explained by the fact that the nervous system function is specific for species, in contrast with more fundamental processes as transcription, for example. Prediction of neural specific TFBSs appears to be harder than others; fortunately, it has been shown that homotypic CRMs are a good predictor of regulatory elements, especially for target genes related to TFs involved in neural development [17]. In a general way, homotypic CRMs are strongly associated with the region surrounding the TSS and the developmental enhancers, with no systematic phylogenetic conservation. These observations rationalize the results we obtained with a single-genome approach and the PPM-based method focused on the co-occurrence of HMX1-BS pairs, in particular with the mouse axon guidance pathway enrichment. Finally, we think that adding a distance range constraint to the PPM gave more accurate results than simply counting the TFBSs in the screening window. Actually, optimal distances for interactions are supposed to be specific for a given TF and including this parameter in the model should give more specific results [26].

In conclusion, our strategy was successful in identifying *HMX1* targets because the PPM we constructed based on the P15 microarray data revealed, a posteriori, an important pathway involved in retinal development (I.E., axon repulsion during retinal axon guidance) in addition to the first three targets (I.E., *Sgcg*, *Tshz2*, *and Slc6a9*). These subsequent outcomes provided additional proof of the robustness of our PPM approach, and open up new opportunities to focus experimental investigations on this specific aspect of the *Hmx1* pathway.

## Appendix 1. Official gene symbols and gene accession numbers of all predicted targets.

To access the data, click or select the words “Appendix 1.”

## Appendix 2. Sequences and amplicon sizes of qPCR primer pairs used in this study.

To access the data, click or select the words “Appendix 2.”

## Appendix 3 Upregulated genes with a FDR<0.1 and a fold-change>1.2.

To access the data, click or select the words “Appendix 3.” ^#^Orientation of HMX1-BSs are indicated; F, forward strand (CAAGTG); R, reverse strand (CACTTG).

## Appendix 4. Downregulated genes with a FDR<0.1 and a fold-change>1.2.

To access the data, click or select the words “Appendix 4.” ^#^Orientation of HMX1-BSs are indicated; F, forward strand (CAAGTG); R, reverse strand (CACTTG).

## Appendix 5. Gene functional classification of FDR<0.1 differentially expressed genes according to DAVID software.

To access the data, click or select the words “Appendix 5.”
